# Case of Dysentery Treated by Injections of Nitrate of Silver

**Published:** 1849-03

**Authors:** 


*Case of Dysentery Treated by Injections of Nitrate of Silver.*—A female, 45 years old, was brought to the Hopital Necker, suffering from dysentery of five days' duration. The symptoms had been much ameliorated by the use of opium and other remedies, but the evacuations were still seven or eight in the day, consisting chiefly of bloody mucus, and accompanied by severe pain.

The following injection was ordered:—Crystallized nitrate of silver, 25 grammes; distilled water, 200 grammes; to be used in the following manner:—After each injection of the solution, another injection of about 300 grammes of tepid water immediately to be given, in order that the nitrate may be conveyed high up into the intestine, and come in contact with a larger extent of surface.

The next day the amelioration was remarkable, There had been three stools which contained much less of the bloody mucus. There was considerable tenesmus. After four days' treatment, the stools became perfectly normal. They contained more mucus, but little tinged with blood. There was only one stool daily, which was not attended with pain. During convalescence, and in consequence of some error of diet, the diarrhœa occasionally returned, the stools being tinged with blood, but without any appearance of mucus; it was, however, always immediately controlled by the injection of the nitrate of silver, followed the next day by a simple injection of starch; and the patient eventually quitted the hospital quite well.

This case not only shows the efficacy of nitrate of silver enemata, but also how they may be used with perfect safety; which fact, for a considerable period, had been denied. A strong solution of the salt has been injected into the rectum and colon, without producing the least accident, either immediately or consecutively. It acts upon the mucous membrane of the parts as it acts upon the lining membrane of the urethra, or the conjunctiva, or upon any other mucus lining to which it is applied.—*Medical Times.*

